# Types of Sliding Calcaneal Osteotomy Fixation: A Systematic Review and Meta-Analysis

**DOI:** 10.7759/cureus.32795

**Published:** 2022-12-21

**Authors:** Samir Hakeem, Hany Elbardecy, Rafee Alnajjar, Wafi Mohammed, Andre McLeod

**Affiliations:** 1 Department of Trauma and Orthopaedics, Galway University Hospital, Galway, IRL; 2 Department of Trauma and Orthopaedics, Cork University Hospital, Cork, IRL; 3 Department of Trauma and Orthopaedics, National Centre for Pelvic and Acetabular Surgery, Tallaght University Hospital, Dublin, IRL; 4 Department of Orthopaedics, Cork University Hospital, Cork, IRL

**Keywords:** fixation, calcaneus, osteotomy, foot and ankle, orthopaedic

## Abstract

Introduction

Different methods are used to fix a sliding calcaneal osteotomy for hindfoot varus and valgus deformity. However, information about the effectiveness and limitations of each method is limited. In this meta-analysis, we compare the hardware removal rate, union rate, and complications of three different methods of fixation: plate, headed screw, and headless screw.

Methods

A systematic review and meta-analysis of published articles were carried out, following the recommendations of the Preferred Reporting Items for Systematic Reviews and Meta-Analysis (PRISMA) guidelines. We investigated diverse databases, Web of Science, PubMed, the Cochrane Library, Excerpta Medica database (EMBASE), and Cumulative Index of Nursing and Allied Health Literature (CINAHL), to search articles reporting the use of different calcaneal osteotomy fixations from database inception to October 2021. The primary outcome was the hardware removal rate, and the secondary outcomes of interest were the union rate and complications.

Results

Of 1,903 articles identified, eight met the inclusion criteria. The highest risk ratio (RR) of the hardware removal rate was detected in the headed screw method (RR: 0.39, 95% confidence interval (CI): 0.26-0.58). However, the highest RR of nonunion was detected in the plate method (RR: 0.02, 95%CI: 0.01-0.07). Regarding complications (infections), the headed screw method presented the highest RR of infection (RR: 0.24, 95%CI: 0.06-0.97).

Conclusion

This comprehensive review and meta-analysis revealed that the headless screw method may be the most effective fixation option for calcaneal osteotomy with the lowest risk of hardware removal rate, nonunion rate, and complications. Obviously, further studies are needed on a larger number of patients to confirm this finding.

## Introduction

Sliding calcaneal osteotomy is a joint-sparing and extra-articular process that is indicated for the surgical correction of planovalgus and cavovarus foot deformity [1]. It is frequently performed in combination with other procedures to better correct global foot deformity. In 1893, Gleich described calcaneal osteotomy for the first time, and then, it was popularized in 1955 by Dwyer [2,3]. Since then, it has been applied in various forms for both varus and valgus correction of the calcaneus and has been used for diverse pathological disorders [4,5].

Historically, many authors believe that osteotomy should be combined with other soft tissue and bony procedures to have good outcomes [6]. A great variety of fixation methods have been reported for calcaneal osteotomy, all of which had comparatively satisfactory results [6-8]. The main concern with these diverse fixation options is appropriate stability for healing to occur without subsequent symptomatic hardware. One potential complication of an unfixed osteotomy is nonunion. Hence, the avoidance of postoperative difficulties, including delayed or nonunion of the osteotomy emplacements and reducing soft tissue destruction, is the most important issue for ankle and foot surgeons [8].

Plate or staple fixation is preferred [7]. Plating is a good option but can lead to peroneal irritation and subsequent removal [9,10].

Painful hardware is relatively frequent for calcaneal osteotomy. Screw heads that are placed in the posteroinferior tuberosity or a lateral plate are relevant causes of soft tissue irritation due to the hardware, requiring subsequent hardware removal. Consequently, patients should be cautioned about potential hardware-related pain and a possible follow-up procedure to remove the hardware.

Understanding the advantages and disadvantages of each fixation option can help in choosing the most appropriate one with the least risk of complications [11].

Therefore, the aim of this systematic review and meta-analysis was to assess the hardware removal rate, union rate, and complications following the fixation of a calcaneal osteotomy with a slide plate, headed screw, and headless screw.

## Materials and methods

Methodology

Study Design and Database Searching

The present systematic review and meta-analysis was carried out following the Preferred Reporting Items for Systematic Reviews and Meta-Analysis (PRISMA) guidelines [12]. The following databases were used to search potentially interesting articles published from database inception to October 2021: Excerpta Medica database (EMBASE), Web of Science, PubMed, the Cochrane Library, and Cumulative Index of Nursing and Allied Health Literature (CINAHL). A systematic search was conducted involving all pairwise combinations of calcaneal osteotomy and the following items: “fixation,” “plate,” and “screw.”

Selection Criteria

Relevant articles were screened by title and abstract after suppressing duplicates. Studies were eligible for inclusion if they addressed the comparison of different fixation methods of calcaneal osteotomies. The remaining studies were then examined in full text to confirm eligibility.

The inclusion criteria for the articles were as follows: (1) retrospective comparative studies, (2) patients who had undergone a sliding calcaneal osteotomy, (3) publications evaluating the hardware removal rate, union rate, and complications described as outcomes, and (4) publications reporting sufficient data regarding complication outcomes.

The exclusion criteria for the studies were as follows: (1) no full text electronically available, (2) publications in a language other than English, (3) letters, editorials, comments, protocols, review papers, and guidelines, and (4) articles with limited outcome information.

Data Extraction

Two independent authors retrieved information from the eligible articles following the inclusion and exclusion criteria, and data were collected on a standardized data sheet that included author name, year, study type, evidence level, geographic origin, sample size, participant sex and age, and outcome ascertainment.

Outcome measures

The primary outcomes were the hardware removal rate and complications, and the secondary outcome was the union rate.

Statistical analyses

RevMan version 5.4 (Cochrane Collaboration, Oxford, United Kingdom) was used to conduct statistical analyses. Odds ratio (OR) with 95% confidence intervals (CIs) was calculated to evaluate all the outcomes. A P value of <0.05 was considered as the level of significance. The Cochrane chi-squared test was conducted to evaluate heterogeneity among articles, with a P value < 0.05 indicating the existence of heterogeneity. To estimate the impact of heterogeneity on the meta-analysis, the I2 value was calculated. Indeed, I2 values ≥ 50% and P < 0.05 indicated a moderate to a high degree of heterogeneity among pooled studies. A fixed-effects design was used in the case of I2 < 50% and P > 0.05; if not, a random-effects design was adopted [13]. We also performed a sensitivity analysis to assess the possible source of heterogeneity. Egger’s test was conducted using the Statistical Package for the Social Sciences (SPSS) version 25 (IBM SPSS Statistics, Armonk, NY, USA) to evaluate publication bias. This latter was further assessed based on the visual inspection of the symmetry in funnel plots.

## Results

Study identification

**Figure 1:**
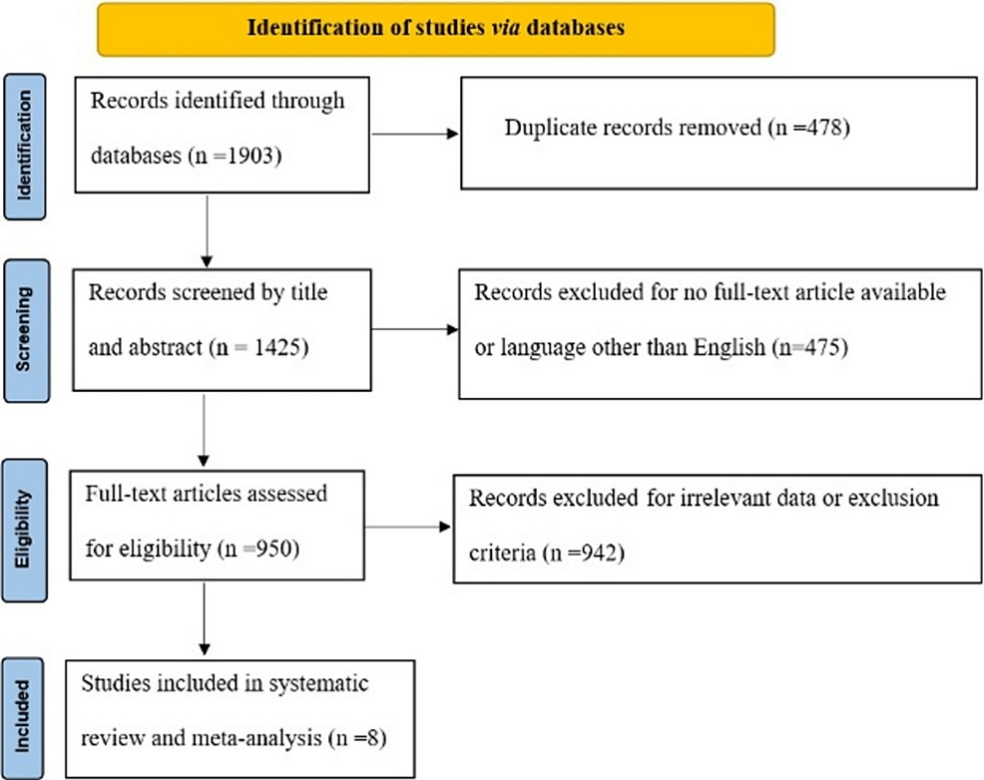
Outline of the PRISMA instructions used to carry out this systematic review and meta-analysis PRISMA: Preferred Reporting Items for Systematic Reviews and Meta-Analysis

Database searching identified 1,903 studies to be screened, of which 950 abstracts were revealed as potentially eligible and retrieved for full-text review. Eligibility criteria were met by eight articles, which were included in this systematic review and meta-analysis. The PRISMA study flowchart is presented in Figure 1.

Features of the Included Articles

The included articles were published between 2013 and 2019. Only one study was conducted in the UK, while the remaining ones were performed in the USA. The included studies presented a high level of evidence (III or IV). The sample size of the included articles varied from 55 to 255. In summary, the total number of participants was 1,062, with 1,092 procedures and a minimum follow-up of three months. The studies’ characteristics are summarized in Table 1.

**Table 1:** Characteristics of the included studies

Article, year	Country	Study design	Level of evidence	Period	Number of patients	Sex	Average age (range)	Number of procedures	Type of fixation (number of procedures)	Average follow-up (range)	Outcomes
Abbasian et al., 2013 [14]	UK	Retrospective case-control study	Level III	2008-2013	55	Male/female ratio: 2:2.5	48 (19-81) years	67	Lateral locking plate (32), headless screw (18), headed screw (17)	32.5 months (3.5-150)	Union rate, hardware removal rate, complication, paresthesia
Haggerty et al., 2019 [15]	USA	Retrospective case series	Level IV	September 2013-December 2018	81	36 males, 45 females	59.7 (18-82) years	81	Slide plate (81)	15 months (4-36)	Hardware removal rate, healing, union rate, complication
Kunzler et al., 2017 [16]	USA	Retrospective study	Level IV	January 2010-December 2014	74	15 males, 59 females	47 years	74	Headless screw (44), headed screw (30)	-	Hardware removal rate, union rate, complication
Lucas et al., 2014 [10]	USA	Retrospective comparative study	Level III	June 2010-January 2012	149	-	-	149	Headed screw (149)	12 months (minimum)	Hardware removal rate
Lucas et al., 2014 [17]	USA	Retrospective comparative study	Level III	June 2010-January2012	228	-	52.2 (36-68) years	228	Headed screw (165), locked lateral compression plate (63)	12 months (minimum)	Hardware removal rate
SahraNavard et al., 2017 [18]	USA	Retrospective study	-	2009-2015	190	112 males, 78 females	48.4 (18-83) years	190	Headless screw (115), headed screw (75)	28 (12-150) weeks	Hardware removal rate, union rates, complications
Saxena et al., 2016 [19]	USA	Cohort study	Level III	November 2007-July 2013	30	13 males, 17 females	55.8 (18-72) years	31	Single screw (17), locking plate (14)	-	Hardware removal rate, union rates, complications
Sayres et al., 2014 [11]	USA	Retrospective case series	Level IV	January 1996-April 2012	255	95 males, 177 females	55 (18-82) years	272	Headed screw (272)	At least two years	Hardware removal rate, union rates

Outcome measures

Hardware Removal Rate

**Figure 2:**
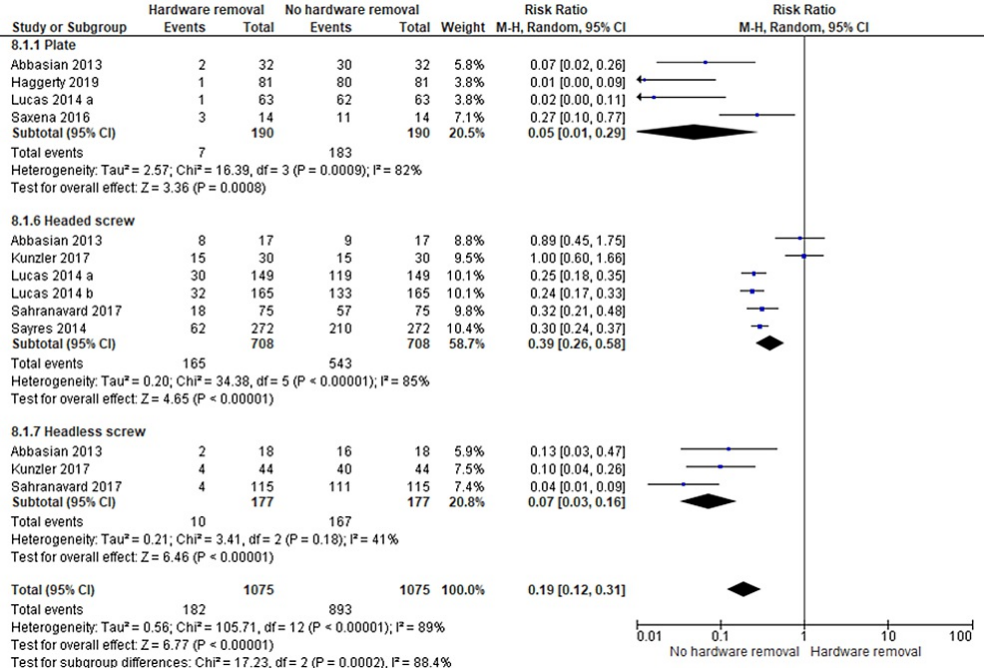
Forest plot representing the risk ratio of hardware removal rate among the three different fixation methods

Among the eight included studies, three different fixation methods were reported: plate, headless screw, and headed screw. The evaluation of the hardware removal outcome was characterized by a high heterogeneity (P < 0.00001, I2 = 89%), so a random-effects model was used (Figure 2). We noticed that the highest risk ratio of the hardware removal rate was detected in the headed screw method (RR: 0.39, 95%CI: 0.26-0.58), while the lowest risk ratio was revealed in the plate method (RR: 0.05, 95%CI: 0.01-0.29). In general, the pooled risk ratio of the hardware removal rate was significantly low (RR: 0.19, 95%CI: 0.12-0.31, P < 0.00001). Consequently, the difference in the hardware removal rate between the techniques was statistically significant (P = 0.0002) (Figure 2).

Union Rate

**Figure 3:**
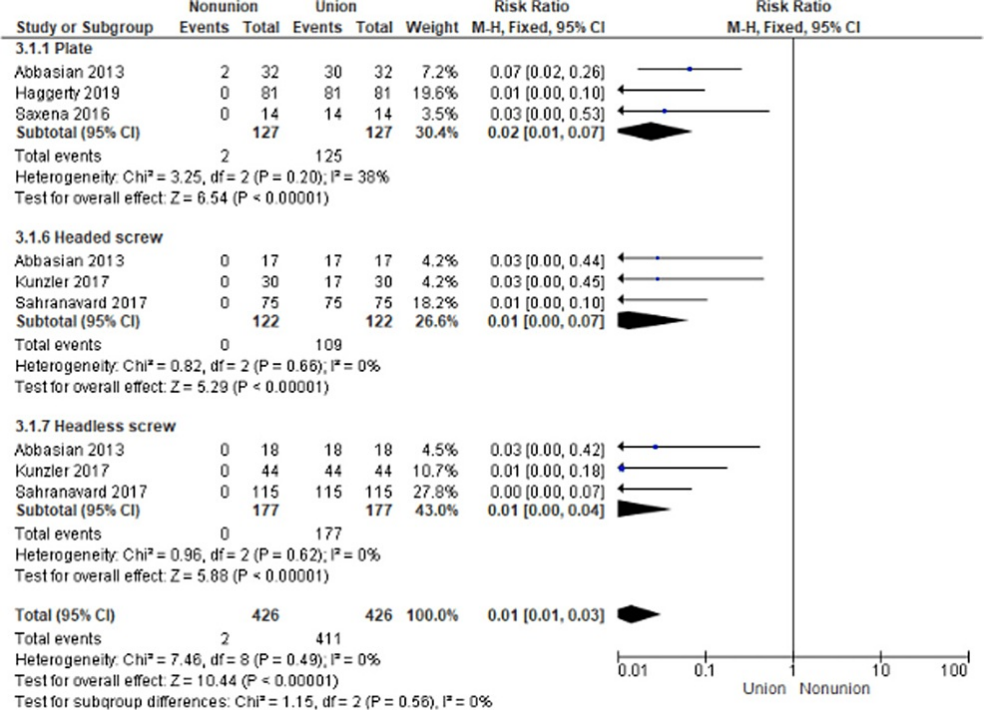
Forest plot representing the risk ratio of the union rate among the three different fixation methods

The evaluation of the union rate outcome was characterized by a low heterogeneity (P = 0.62, I2 = 0%), so a fixed-effects model was used (Figure 3). We noticed that the highest risk ratio of nonunion was detected in the plate method (RR: 0.02, 95%CI: 0.01-0.07), while the lowest risk ratio was revealed in both screw methods (RR: 0.01, 95%CI: 0.00-0.04). In general, the pooled risk ratio of the nonunion rate was significantly low (RR: 0.01, 95%CI: 0.01-0.03, P < 0.00001). However, the difference in the nonunion rate between the techniques was not statistically significant (P = 0.56) (Figure 3).

Infections

**Figure 4:**
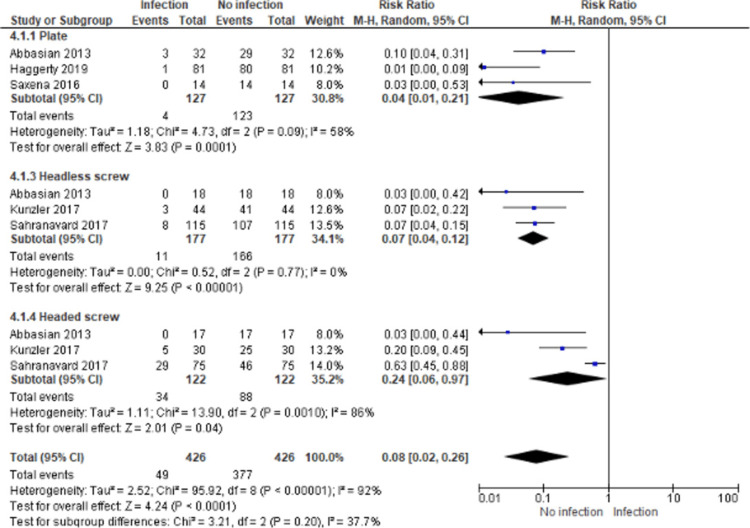
Forest plot representing the risk ratio of infection among the three different fixation methods

The evaluation of the infection outcome was characterized by a high heterogeneity (P = 0.001, I2 = 86%), so a random-effects model was used (Figure 4). We noticed that the highest risk ratio of infection was detected in the headed screw method (RR: 0.24, 95%CI: 0.06-0.97), while the lowest risk ratio was revealed in the plate method (RR: 0.04, 95%CI: 0.01-0.21). In general, the pooled risk ratio of infection was significantly low (RR: 0.08, 95%CI: 0.02-0.26, P < 0.00001). However, the difference in the infection rate between the techniques was not statistically significant (P = 0.2) (Figure 4).

Sensitivity analysis

Additionally, to further reveal the likely origin of heterogeneity among the hardware removal outcome, a leave-one-out sensitivity analysis was performed. This outcome did not differ markedly, which indicates that the meta-analysis had strong reliability. We revealed that the risk ratio of hardware removal varied between 0.03 (95%CI: 0.01-0.09) and 0.08 (95%CI: 0.01-0.47) in the plate method, between 0.31 (95%CI: 0.23-0.42) and 0.44 (95%CI: 0.27-0.71) in the headed screw method, and between 0.06 (95%CI: 0.02-0.17) and 0.11 (95%CI: 0.05-0.23) in the headless screw method (Table 2).

**Table 2:** Sensitivity analyses of risk ratio in terms of hardware removal rate in plate, headed, and headless screw fixation methods CI: confidence interval Sources: [10,11,14-19]

Study excluded	Risk ratio (95%CI)	Heterogeneity		
Plate				
Abbasian et al. (2013)	0.04 (0.00-0.65)	Chi^2^ = 17.44	P = 0.0002	I^2^ = 89%
Haggerty et al. (2019)	0.08 (0.01-0.47)	Chi^2^ = 10.18	P = 0.006	I^2^ = 80%
Lucas et al. (2014)	0.07 (0.01-0.51)	Chi^2^ = 12.44	P = 0.002	I^2^ = 84%
Saxena et al. (2016)	0.03 (0.01-0.09)	Chi^2^ = 2.88	P = 0.24	I^2^ = 31%
Headed screw				
Abbasian et al. (2013)	0.34 (0.23-0.50)	Chi^2^ = 24.93	P < 0.00001	I^2^ = 84%
Kunzler et al. (2017)	0.31 (0.23-0.42)	Chi^2^ = 12.08	P = 0.0005	I^2^ = 92%
Lucas et al. (2014)	0.43 (0.27-0.70)	Chi^2^ = 31.91	P < 0.00001	I^2^ = 87%
Lucas et al. (2014)	0.44 (0.27-0.71)	Chi^2^ = 30.50	P < 0.00001	I^2^ = 87%
SahraNavard et al. (2017)	0.41 (0.25-0.66)	Chi^2^ = 34.44	P < 0.00001	I^2^ = 88%
Sayres et al. (2014)	0.42 (0.25-0.73)	Chi^2^ = 33.91	P < 0.00001	I^2^ = 88%
Headless screw				
Abbasian et al. (2013)	0.06 (0.02-0.17)	Chi^2^ = 2.37	P = 0.12	I^2^ = 58%
Kunzler et al. (2017)	0.06 (0.02-0.22)	Chi^2^ = 2.40	P = 0.12	I^2^ = 58%
SahraNavard et al. (2017)	0.11 (0.05-0.23)	Chi^2^ = 0.07	P = 0.79	I^2^ = 0%

Publication bias

**Figure 5:**
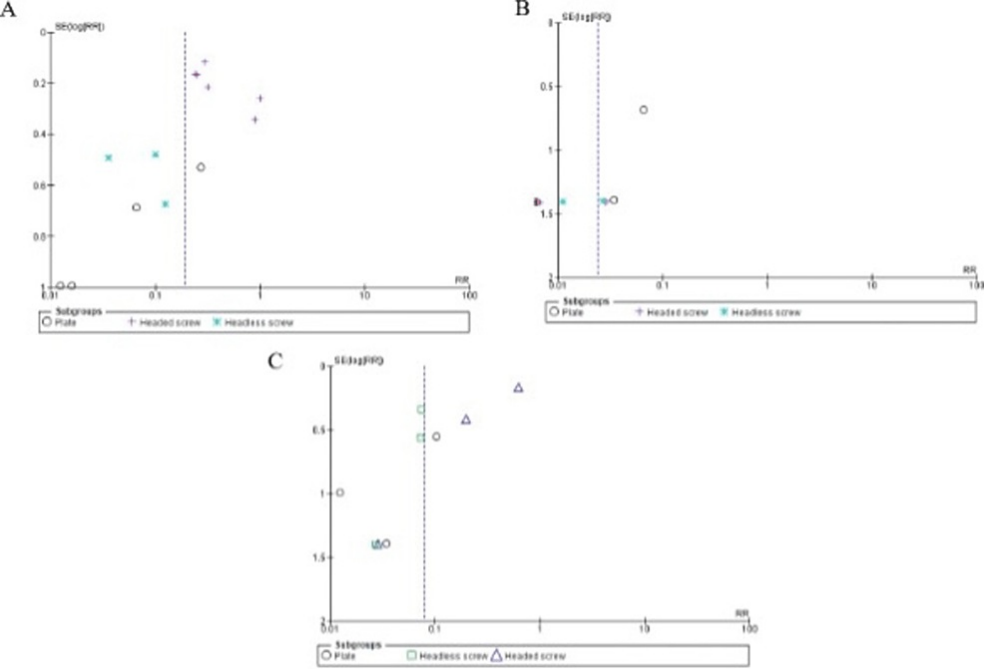
Funnel plots demonstrating no evidence of publication bias among the included studies in terms of (A) hardware removal, (B) union, and (C) infection rates

We demonstrated no proof of publication bias for hardware removal, union, and infection rates using Egger’s regression test (P = 0.59, P = 0.43, and P = 0.48, respectively). Moreover, a visual inspection of the funnel plot revealed a symmetrical funnel (Figure 5).

## Discussion

Calcaneal osteotomy is a powerful procedure frequently used by foot and ankle surgeons for the correction of hindfoot deformities such as cavovarus or pes planovalgus. To the best of our knowledge, this systematic review and meta-analysis study is the first that compares three diverse fixation options for a calcaneal osteotomy.

Hardware irritation inducing subsequent removal of the implant is a common risk of calcaneal osteotomy fixations, and reported removal rates are high in the literature, varying from 6% to 50% [14-16,20]. In this meta-analysis, the prevalence of hardware removal was 39% when using headed screws. Bolt et al. reported a similar result when headed screws were concerned, with a removal rate as high as 53% [21]. Similar results were cited in another work that reported the use of two headed screws for pes planovalgus corrections [22]. At an average of 12 months post-surgery, the authors detected a 35% rate of hardware removal. It could be explained by the fact that the subcutaneous protrusion created over the heel associated with the head of the headed screws may cause headed screws to be removed more frequently than headless screws. It was shown that smaller screws required removal much less frequently than larger ones and patients treated with smaller screws were, therefore, subjected to less potential complications [10]. However, information regarding this issue is limited and conflicting. Interestingly, a significant decrease in the risk of hardware removal was revealed when a plate or a headless screw was used. The removal rate was slightly less in the plate compared to the headless screw groups (5% versus 7%); this may be explained by the fact that an incision over the weight-bearing aspect of the heel is avoided altogether in the plate cases, whereas when using a headless screw, scar tissue at the screw entry site may result in symptoms. Furthermore, it was revealed that one patient remained partially symptomatic over the scar despite the removal of their headless screws. Consequently, we suggest that the use of lateral plates or headless screws may significantly reduce the risk of hardware removal.

Contradictory to hardware removal, calcaneal osteotomies have high union rates, which is due to the cancellous nature and the vascularity of the bone. Our results are consistent with the current literature, which shows a high overall union rate following calcaneal osteotomies and reaching 98% for plate and 99% for both headed and headless screws. This finding is in line with previous studies. Indeed, Wacker et al. [23] and Maskill et al. [24] reported no cases of nonunion in their studies, while Bolt et al. [21].​​ detected only one nonunion in 17 (6%) cases that required bone grafting. The calcaneal osteotomy has been fixed using a single screw only [21], while others used double screws [10,18]. In both cases, the union rate is similarly high. Similar results have also been detected with small and large screws [10], suggesting that the number and size of screws used do not appear to affect the union rate.

Regarding complications, wound infection is the most frequent complication detected occurring in calcaneal osteotomy fixation. This meta-analysis showed that the overall rate of wound infection is relatively low among the three techniques (8%), which is comparable with the other series. However, headed screws presented the highest rate of infection (24%). Some studies reported other types of complications. A 12% rate of minor paresthesia was detected in the study of Abbasian et al. [14], but no cases of injury to the branches of the sural nerve or sural neuritis were revealed. Two previous studies have reported sural neuritis and neuroma in 2%-6% of cases [23,24]. In both studies, further surgery and exploration were undertaken for the one-third of these cases to address their symptoms.

Hardware removal procedures, delayed union or nonunion, and wound infections can have significant adverse quality of life implications for the patient, leading to extended activity limitations, work time lost, and increased healthcare expenditures [23-25]. Consequently, foot and ankle surgeons should carefully consider their choice of fixation option following calcaneal osteotomy because this later may require subsequent surgery for the removal of hardware.

Limitations

Several limitations in this systematic review and meta-analysis need to be taken into account. Firstly, although we conducted the search process in five different databases, the number of included studies was limited. Secondly, we revealed an elevated rate of heterogeneity between the articles; what causes major difficulties was the size of all available studies. Hence, considerable heterogeneity, which is expected in meta-analysis studies, can alter the interpretability of results [26]. Consequently, the findings of the present work have to be analyzed with attentiveness. Finally, there is a lack of standardization among the patients with respect to their deformities, operative procedure, and general medical conditions. The number of patients involved in the various fixation options were also small secondary to the relatively stringent inclusion criteria and limited data available in published reports. A larger, randomized, standardized study would be the gold standard to make any firm conclusions.

## Conclusions

To summarize, 39% of patients underwent hardware removal and 24% suffered from infections when a headed screw was used. The use of headless screws or plates significantly reduced these risks. The use of plates was associated with a slightly higher rate of nonunion. Headless screws achieved the best results and the lowest complication rates in this systematic review and meta-analysis. It must, however, be noted that the numbers of studies involved were small, and firm conclusions can only be drawn from a larger randomized series.
